# True ileal digestibility of legumes determined by dual-isotope tracer method in Indian adults

**DOI:** 10.1093/ajcn/nqz159

**Published:** 2019-08-02

**Authors:** Sindhu Kashyap, Aneesia Varkey, Nirupama Shivakumar, Sarita Devi, Rajashekar Reddy B H, Tinku Thomas, Thomas Preston, Sheshshayee Sreeman, Anura V Kurpad

**Affiliations:** 1 Division of Nutrition, St. John's Research Institute, St. John's National Academy of Health Sciences, Bangalore, India; 2 Department of Physiology, St. John's Medical College, St. John's National Academy of Health Sciences, Bangalore, India; 3 Department of Biostatistics, St. John's Medical College, St. John's National Academy of Health Sciences, Bangalore, India; 4 Scottish Universities Environmental Research Centre, East Kilbride, Scotland, United Kingdom; 5 Department of Crop Physiology, University of Agricultural Sciences, Bangalore, India

**Keywords:** intrinsic labeling, dual-isotope tracer technique, legume protein, chickpea, yellow pea, mung bean, dehulling, digestible indispensable amino acid score, DIAAS

## Abstract

**Background:**

Good-quality plant protein sources are important for protein adequacy in a balanced diet. Legumes are known to be a source of good quality plant protein, but the true ileal digestibility of indispensable amino acids (IAAs) of commonly consumed legumes is not known in humans.

**Objectives:**

In this study we measured the true ileal IAA digestibility of ^2^H-intrinsically labeled chickpea, yellow pea, and mung bean (hulled and dehulled) protein, using the dual-isotope tracer technique referenced to a standard protein ([U-^13^C] spirulina). The study also aimed to validate the use of [U-^13^C] spirulina as a reference protein in this method.

**Methods:**

^2^H-intrinsically labeled legumes, obtained by watering plants with deuterium oxide (^2^H_2_O), were administered in a plateau feeding method to healthy Indian adults to measure their true ileal IAA digestibility with the dual-isotope tracer technique, using [U-^13^C] spirulina protein or a ^13^C-algal IAA mixture as the standard.

**Result:**

The true ileal IAA digestibilities (mean ± SD) of chickpea, yellow pea, and mung bean were 74.6 ± 0.8%, 71.6 ± 1.3%, and 63.2 ± 1.5%, respectively. The true mean ileal IAA digestibility of mung bean when referenced to [U-^13^C] spirulina protein or a ^13^C-algal IAA mixture did not differ significantly (63.2 ± 1.5% versus 64.0 ± 2.4%, *P* > 0.05). The true ileal IAA digestibility of mung bean improved to 70.9 ± 2.1% after dehulling.

**Conclusions:**

The true mean ileal IAA digestibility of legumes in healthy Indian adults was lower than expected. Traditional processing techniques such as dehulling improve protein digestibility by about 8%. This study was registered in the Clinical Trials Registry of India (CTRI): CTRI/2017/11/010468 (http://ctri.nic.in, accessed on 28/03/2019).

## Introduction

The risk of protein inadequacy is known to be higher in low- and middle-income countries (LMICs) and to be associated with food insecurity, dietary patterns of the population, and influence of environmental factors such as infections (1, 2). An additional dimension of the protein requirement is its quality, which is a product of the protein's digestibility and its indispensable amino acid (IAA) composition. Animal source foods (ASFs), such as milk, eggs, and meat, and plant source foods (PSFs), such as legumes, have high- and good-quality proteins, respectively, with a high lysine content, and the appropriate addition of these foods into a cereal-based diet, which is known to be limited in lysine, is of particular importance in populations consuming such diets (1). However, environmental and economic costs are also important considerations in planning future optimal diets. For example, the lifecycle environmental impact of a nonvegetarian meal has been shown to be 2 or more times higher than that for a vegetarian meal (3, 4). A recent report has also suggested that the quantity of PSF should increase and that of ASF decrease in a healthy plate to achieve improved health and environmental sustainability (5).

Legumes are an important source of good-quality protein in plants and therefore important for vegetarian populations. However, the digestibility of legumes is lower than that of ASF protein in rodent models (6–9) using orofecal balances, which could overestimate digestibility, and hence legume digestibility might be much lower than reported. A recent FAO report recommended that true protein digestibility should be measured at the end of the ileum as an oro-ileal balance (10) because colonic bacteria can recycle urea and confound orofecal balance measurements and there is no significant IAA absorption from the colon. Further, this report recommended that digestibility should be measured for each IAA, since each could have a different true oro-ileal digestibility (10). Conceptually, these factors are embedded in a new index of protein quality called the digestible indispensable amino acid score (DIAAS) (11).

The accurate estimation of the true ileal digestibility of legume protein is critical to determining the optimal dietary intake, particularly when the ASF intake is low for economic or cultural reasons. Recently, a minimally invasive dual-isotope technique has been developed to determine the true ileal IAA digestibility of food proteins in humans (12). In this technique, the appearance of labeled amino acids in plasma from intrinsically labeled food protein (^13^C or ^2^H) is compared to that of a simultaneously ingested but differently labeled standard protein of known digestibility.

This study therefore primarily aimed to measure the digestibility of 3 commonly consumed legumes (chickpea, yellow pea, and mung bean) using the dual-isotope tracer technique as described (12) against a standard of [U-^13^C]spirulina protein. Secondarily, it aimed to validate the use of the [U-^13^C]spirulina protein standard in the dual-tracer method against a standard of a ^13^C-algal IAA mixture and to measure the true ileal IAA digestibility of mung bean after dehulling, since this is affected by intrinsic antinutritional factors (ANFs) present in the hull of the legume (13).

## Methods

### Intrinsic ^2^H labeling of the legumes chickpea, yellow pea, and mung bean

The commonly consumed legumes chickpea, yellow pea, and mung bean were selected for determination of the true ileal IAA digestibility of legumes. The kabuli chickpea (*Cicer arietinum*; garbanzo bean; var. KAK-2) and the mung bean (*Vigna radiata*; var. KKM-3) were grown in the winter (rabi) and rainy (kharif) season for 2 consecutive y at the University of Agricultural Sciences, Bengaluru, India. A standardized labeling protocol using deuterium oxide (^2^H_2_O, 99.9%, Sercon Ltd.) was followed as previously described in detail (12) and briefly mentioned here. The seeds were sown in 20-L pots after the soil conditions were optimized (farmyard manure ratio and water requirements). The plants were grown under a rainout shelter and irrigated per requirement during the initial vegetative growth phase. A gravimetric irrigation protocol, started 1 wk before the stable isotope labeling, helped in determining the water requirements to attain 50% and 70% field capacity for chickpea and mung bean, respectively, which were maintained thereafter. The ^2^H_2_O dosing was timed to seed development and started at the 50% flowering stage. An initial bolus of 400 mL (25% ^2^H_2_O) on the 1st day followed by 100-mL pulses of 2.5% ^2^H_2_O on the 3rd, 5th, 7th, and 9th days were applied to the pots along with the irrigation water. The yellow pea (*Pisum sativum*; var. Salamanca) was sown in spring and harvested in summer in a greenhouse at the James Hutton Institute. The yellow peas were grown in 60-L troughs containing an optimum soil-to-compost ratio and maintained at 90% field capacity using an automated irrigation system. At flowering, each trough received a single bolus of 450 mL 25% ^2^H_2_O (Sigma Aldrich), and for the next 14 d the plants were irrigated with 2% ^2^H_2_O. A D3 Dosatron unit having an adjustable injection rate of 0.2–2% was used to dispense ^2^H_2_O constantly to the irrigation water. The seeds were harvested at maturity, air dried, pooled, and stored for analysis and human experimentation. A subsample of the pooled seeds was ground to fine flour using a mortar and pestle and subjected to acid hydrolysis, cation exchange cleanup, and derivatization before estimation of ^2^H enrichments in IAAs using GC pyrolysis–isotope ratio MS (GC-P-IRMS; Delta V Advantage, Thermo Fisher Scientific Inc.) as described previously (12).

### Human legume digestibility protocol

For the measurement of true ileal IAA digestibility of the selected legumes, using the dual-tracer method with intact [U-^13^C]spirulina protein as standard, a local habitually consumed recipe of ghee rice and tomato curry was standardized as a base meal to which 1 of the selected legumes (chickpea, yellow pea, and whole mung bean) was added. The test meal provided one-third of the daily energy and protein requirements for adults, with legumes contributing two-thirds of the total protein from the meal. The nutrient composition of the test meals in each legume group is provided in **Table 1**. The chickpea, whole mung bean, and yellow pea were soaked overnight for 12 h and pressure cooked, using the water remaining at the end of soaking, for 15 min on the morning of the experiment day. The prepared meal aliquots (see below) were warmed for 10 s in a microwave oven before being administered to the subjects. The [U-^13^C]spirulina (12 mg/kg, Cambridge Isotope Laboratories; 97% purity), a standard protein source with predetermined true ileal IAA digestibility, was mixed into each meal (12).

**TABLE 1:** Nutrient composition of the standardized test meal for each test protein group^1^

	Test protein				
Nutrient	CP	YP	MB	MB-^13^CAA	DHMB
Energy, kcal	834.5 ± 90.2	812.4 ± 22.0	735.1 ± 76.2	745.6 ± 101.2	749.5 ± 88.0
Protein, g	21.5 ± 2.8	24.0 ± 2.8	22.6 ± 2.1	23.7 ± 2.7	23.6 ± 2.3
Fat, g	26.5 ± 3.9	22.2 ± 0.8	21.4 ± 2.0	22.8 ± 2.9	22.4 ± 2.1
Carbohydrate, g	125.7 ± 9.4	125.6 ± 2.8	111.0 ± 12.5	109.9 ± 16.7	111.1 ± 14.7
P/E ratio	10.3 ± 0.3	12.4 ± 0.1	12.3 ± 0.3	12.7 ± 0.3	12.6 ± 0.3

1 Values are means ± SDs. Subjects were different in CP, YP, and MB digestibility studies (*n* = 3 each male and female). Paired studies for MB versus DHMB (*n* = 6) and MB versus MB-^13^CAA (*n* = 5, *n* = 3 female and *n* = 2 male). Subjects of MB-^13^CAA study were subsets of the MB study. CP, chickpea; DHMB, dehulled mung bean; MB, mung bean; MB-^13^CAA, mung bean true ileal digestibility referenced to standard ^13^C IAA mixture; P/E, protein/energy; YP, yellow pea.

For the validation of the use of [U-^13^C]spirulina protein as standard, digestibility of whole mung bean was remeasured using a ^13^C-algal IAA mixture (1.25 mg/kg, Sigma Aldrich; 98% purity) as the standard protein, in the same subjects in whom digestibility had been measured (in the first set of experiments) using intact [U-^13^C]spirulina protein as the standard protein. The composition of the ^13^C–algal IAA mixture [which included dispensable amino acid (AA)] is given in **Supplemental Table 1**. To study the effect of the legume hull ANF on digestibility, mung beans were manually dehulled after being soaked for 12 h, stored at −20^⁰^C, and then thawed and pressure cooked for 12 min on the morning of the experiment. The legumes were analyzed for their protein, IAA, fiber, and polyphenol content by a commercial analytical laboratory (Eurofins Analytical Services). Raw and cooked legumes were ground to fine flour before the analysis. For raw legumes, raw seed flour was produced by dry grinding; for cooked legumes, these were soaked, pressure cooked, homogenized, and then lyophilized. The protein content of raw and cooked legume flour was analyzed by the Kjeldahl method (14) with a precision of ± 5%. The IAA profile was analyzed after hydrolysis of the raw and cooked legume flour in 6 M HCl at 115^⁰^C for 24 h. Amino acids were derivatized with 6-aminoquinolyl-*N*-hydroxysuccinimidyl carbamate (AccQ.Fluor reagent kit, Waters) and analyzed by HPLC with fluorescence detection (1220 Infinity LC, Agilent). The assay used a standard reference material (1869, NIST) as a quality control, and its precision was within ± 10%. Dietary fiber was quantified using the AOAC method (15), and polyphenols were analyzed by the Folin Ciocalteu method (16) in cooked legumes; the precision for both of these assays was within ± 10%.

Healthy subjects of both sexes between 18 and 45 y old with normal BMI of 18.5–25 kg/m^2^ (*n* = 3 each female and male for all legumes with the [U-^13^C] spirulina protocol; and paired *n* = 3 female and *n* = 2 male for the validation study with the ^13^C-algal IAA mixture; 1 subject could not participate in the repeat validation study), and those who did not have food allergies were recruited. Pregnant women, individuals with current history of smoking, being on iron supplements, antibiotic usage within 4 wk prior to the experiment, and those with serious medical or surgical history in the past 3 mo or with current acute illness were excluded. Details of the subject screening and enrollment are provided in **Supplemental Figure 1**. The institutional ethical review board approved the study and informed consent was obtained from all subjects. Subject characteristics at baseline for each experimental protocol are provided in **Table 2**.

**TABLE 2:** Demographic and anthropometric characteristics of study participants in each test protein group^1^

	Test protein			
Variable	CP	YP	MB-^13^CAA	MB/DHMB
Age, y	23.8 ± 2.2	22.8 ± 3.5	24.6 ± 3.9	23.5 ± 3.8
Weight, kg	58.0 ± 10.0	56.3 ± 2.7	56.3 ± 5.0	55.5 ± 4.3
Height, m	1.6 ± 0.1	1.6 ± 0.1	1.6 ± 0.1	1.6 ± 0.1
BMI, kg/m^2^	22.2 ± 1.9	21.3 ± 2.5	20.8 ± 1.4	20.5 ± 0.9
Hemoglobin, g/dL	13.3 ± 1.7	13.8 ± 1.5	12.4 ± 1.8	12.4 ± 1.6

1 Values are means ± SDs. Subjects were different in CP, YP, MB digestibility studies (*n* = 3 each male and female). Paired studies for MB versus DHMB (*n* = 6) and MB versus MB-^13^CAA (*n* = 5, *n* = 3 female and *n* = 2 male). Subjects of the MB-^13^CAA study were a subset of the subjects of the MB study. CP, chickpea; DHMB, dehulled mung bean; MB, mung bean; MB-^13^CAA, mung bean true ileal digestibility referenced to standard ^13^C IAA mixture; YP, yellow pea.

On the day of the experiment, subjects reported at 0630 to the metabolic unit of St. John's Medical College, Bangalore, India, after an overnight fast of 10 h. The experiment started at 0700 and a plateau feeding protocol of 8 h was adopted. The test meal was portioned into 11 parts, each part constituting 1 mini-meal. The first meal constituted a priming meal (3 mini-meals combined), to which a prime of ^13^C-bicarbonate (4 mg/kg, Cambridge Isotope Laboratories; > 99% purity) was added, followed by a single mini-meal every hour for the next 7 h. One mini-meal portion was retained for isotopic enrichment analysis. Breath and blood samples were collected during the protocol just before the mini-meal was fed. The breath samples were collected using modified breath bags, transferred and stored in 10-mL glass exetainers (Becton Dickinson) at room temperature until analysis. The breath samples were obtained at baseline followed by hourly samples for the total experimental duration. Blood was collected after securing an indwelling venous catheter (Jelco 22 G, Medex Medical Ltd.) at the beginning of the experiment. A blood sample at baseline was followed by samples collected between hour 5 and 8 of the experiment, representing a plateau period (12). Whole blood was transferred into EDTA-coated evacuated tubes (Becton Dickinson) and centrifuged at 1098 × *g* at 4^⁰^C for 10 min to separate the plasma, which was aliquoted into 1.8 mL cryovials (Cryochill, Tarsons) and stored at −80^⁰^C until analysis.

Breath samples were analyzed for ^13^CO_2_ enrichments using IRMS (Delta V Advantage, Thermo Fisher Scientific Inc.). Plasma samples were deproteinized and amino acids were collected after cation exchange, derivatized to their ethoxycarbonyl ethyl esters (ECEE), and analyzed by LC–MS/MS (6495 iFunnel Triple Quadrupole, Agilent) and GC-P-IRMS (Delta V Advantage), to measure [^13^C] and [^2^H] isotopic enrichments of the IAA, respectively, over basal plasma samples, and expressed as parts per million excess (ppme) (12). Whole-meal samples underwent gas phase acid hydrolysis prior to the measurement of [^13^C] and [^2^H] isotopic enrichments of the IAA in the meal, expressed as ppme (over baseline); for the baseline enrichment of the meal, the same meal was cooked with unlabeled legumes and analyzed (12).

The true ileal IAA digestibility (percentage) of legumes (including dehulled mung bean) was calculated in reference to a standard protein, [U-^13^C] spirulina, using the following equation:

(1) $$\begin{equation*}
\left[ {{\rm{Plasma}}{{\rm{ }}^2}{\rm{H}\hbox{-}\rm{IAA}}\left( {{\rm{ppme}}} \right)/{\rm{Meal}}{{\rm{ }}^2}{\rm{H{\hbox{-}} IAA}}\left( {{\rm{ppme}}} \right)} \right]/\nonumber\\
\quad\left[ {{\rm{Plasma}}{{\rm{ }}^{13}}{\rm{C{\hbox{-}} IAA}}\left( {{\rm{ppme}}} \right)/{\rm{Meal}}{{\rm{ }}^{13}}{\rm{C{\hbox{-}} IAA}}\left( {{\rm{ppme}}} \right)} \right]\nonumber\\
\qquad *100*{\rm{Di}}{{\rm{g}}_{{\rm{Std}}}}/100*{\rm{TCF}}
\end{equation*}$$

where Dig_Std_ is the percentage of true ileal digestibility of IAA of spirulina protein with reference to a standard of a crystalline IAA mixture, measured previously in similar subjects using the same method (12). The mean value of spirulina IAA digestibility was 85.2%, and the Dig_Std_ values used for each amino acid were 84.1%, 95.3%, 82.5%, 77.5%, 86.0%, 84.2%, and 87.1% for methionine, phenylalanine, threonine, lysine, leucine, isoleucine, and valine, respectively (12). A transamination correction factor (TCF) was used to correct for the maximum possible loss of α-^2^H during transamination as described previously (12).

The true ileal IAA digestibility (percentage) of mung bean, when referenced to the standard of a ^13^C-algal free AA mixture (for validation), was calculated using the following equation:

(2) $$\begin{equation*}
\left[ {{\rm{Plasm}}{{\rm{a}}^2}{\rm{H\hbox{-}}\rm{ IAA}}\left( {{\rm{ppme}}} \right)/{\rm{Meal}}{\,\,^2}{\rm{H\hbox{-} IAA}}\left( {{\rm{ppme}}} \right)} \right]/\nonumber\\
\quad\left[ {{\rm{Plasma}}{{\rm{ }}^{13}}{\rm{C\hbox{-} IAA}}\left( {{\rm{ppme}}} \right)/{\rm{Meal}}{{\rm{ }}^{13}}{\rm{C\hbox{-} IAA}}\left( {{\rm{ppme}}} \right)} \right]\nonumber\\
\qquad*100*{\rm{TCF}}
\end{equation*}$$

Note that the Dig_Std_ term is not present and other acronyms are the same as described above.

### Statistical analysis

Data are presented as means ± SDs. The number of subjects selected for each legume study was based on an earlier study on the digestibility of spirulina protein, where the SD of IAA ileal digestibility was 5.4% (12). Based on this finding, 6 subjects per group were required to detect a significant difference in digestibility of 10% with a power of 80%. Since the mean and median of the true ileal digestibility and the individual IAA digestibility values were similar and the SDs were low (less than half the value of the mean), the data were assumed to be normally distributed. An ANOVA was performed to determine significant differences in mean and specific IAA true ileal digestibility between the legumes (chickpea, yellow pea, and mung bean). Differences between specific pairs of legumes were evaluated using post hoc tests with Bonferroni correction. A paired t test was used to evaluate differences in true ileal specific IAA digestibility of whole mung bean measured using [U-^13^C] spirulina or the ^13^C-algal AA mixture as standard. A paired t test was also used to evaluate differences between the IAA digestibility of whole and dehulled mung bean. Comparisons were made of specific differences in IAA digestibility between legumes and between whole and dehulled mung bean, as well as whole mung bean measured with either the [U-^13^C] spirulina or ^13^C-algal AA mixture standard, by adjustment for multiple comparisons of 7 different specific IAA using Bonferroni corrected *P* values. For all the comparisons, *P* < 0.05 was considered significant. All calculations were performed using STATA (version 15.1, StataCorp LLC).

## Results

**FIGURE 1:**
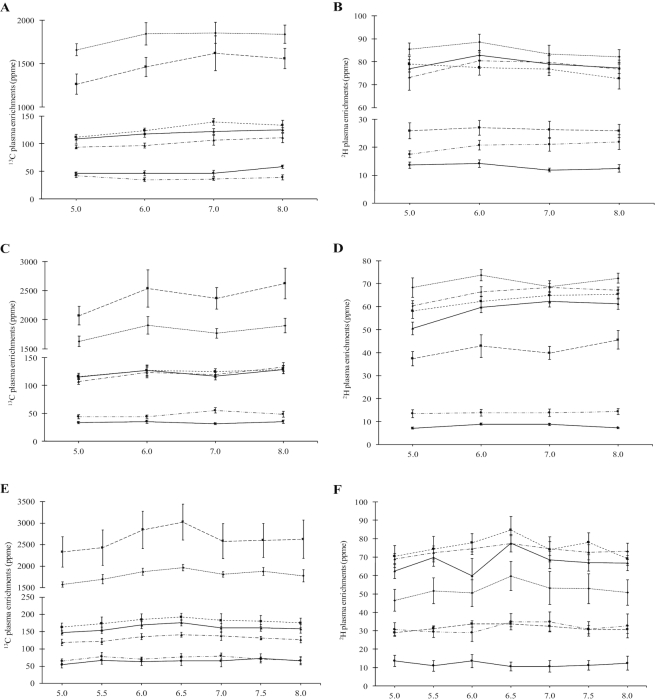

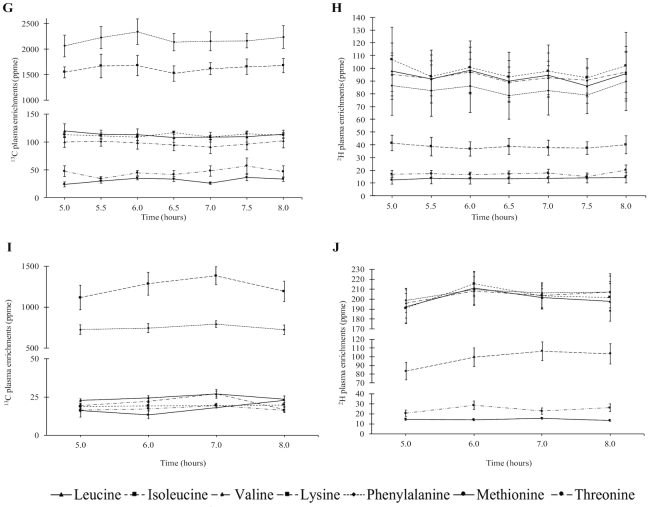
Plasma enrichment of IAA after consumption of different test protein meals. (A) Plasma appearance of ^13^C and (B) ^2^H isotopic enrichments of IAA (ppme) at plateau after consumption of intrinsically labeled *kabuli* chickpea (n = 6); (C) Plasma appearance of ^13^C and (D) ^2^H isotopic enrichments of IAA (ppme) at plateau after consumption of intrinsically labeled yellow pea (n = 6); (E) Plasma appearance of ^13^C and (F) ^2^H isotopic enrichments of IAA (ppme) at plateau after consumption of intrinsically labeled whole mung bean (n = 6); (G) Plasma appearance of ^13^C and (H) ^2^H isotopic enrichments of IAA (ppme) at plateau after consumption of intrinsically labeled dehulled mung bean (n = 6); (I) Plasma appearance of ^13^C and (J) ^2^H isotopic enrichments of IAA (ppme) at plateau after consumption of intrinsically labeled whole mung bean referenced to ^13^C-algal indispensible amino acid mixture (n = 5). Plots represent mean ± SE of ^13^C and ^2^H isotopic enrichments appearance in plasma. IAA, indispensable amino acid; ppme, parts per million excess.

The demographic and anthropometric characteristics of the subjects studied for each test protein are given in Table 2. All subjects were healthy with normal BMI and had no illnesses or infection within 3 mo prior to the study. The total protein content and IAA profile of raw and cooked legumes, and the dietary fiber and total polyphenol content of cooked legumes, are provided in **Table 3**. The mean ^2^H IAA enrichments in pooled raw chickpea, yellow pea, and mung bean were 694, 745, and 1534 ppme, respectively; the enrichment of IAAs of the pooled samples is shown in **Supplemental Table 2**. The mean ± SD (ppme) of ^2^H and ^13^C IAA enrichments within each test meal are given in **Supplemental Table 3**. The ^2^H and ^13^C plasma enrichments of each IAA at plateau (from hour 5 to 8 of the experimental protocol) are shown in **Figure 1**. The mean interindividual CVs of ^2^H and ^13^C plasma enrichment at plateau were 25% and 21%, respectively. The CVs for ^2^H plasma enrichments ranged from 17% for valine to 35% for methionine and the ^13^C plasma enrichments ranged from 15% for lysine to 29% for methionine. The plateau breath ^13^CO_2_ enrichments for all 4 test proteins are shown in **Supplemental Figure 2**. For all the experiments, the priming dose of ^13^C-bicarbonate was the same, and at plateau, the ^13^CO_2_ enrichments in the breath were also similar, indicating a similar oxidative disposal of legume amino acids.

**TABLE 3:** Total protein, IAA profile, dietary fiber, and total polyphenol content of chickpea, yellow pea, and whole and dehulled mung bean^1^

	Raw				Pressure cooked			
Variable	CP	YP	MB	DHMB	CP	YP	MB	DHMB
Protein	22.26	24.25	24.02	25.74	22.93	24.43	24.94	25.17
IAA								
Methionine	0.14	0.19	0.20	0.24	0.24	0.20	0.21	0.23
Phenylalanine	1.33	1.18	1.51	1.60	1.34	1.23	1.54	1.56
Threonine	1.52	1.71	1.20	1.32	1.52	1.56	1.28	1.30
Lysine	1.37	1.57	1.65	1.72	1.44	1.57	1.61	1.61
Leucine	1.50	1.58	1.72	1.84	1.55	1.62	1.75	1.80
Isoleucine	0.97	1.02	0.99	1.14	1.01	1.00	1.05	1.03
Valine	0.96-	1.12	1.17	1.33	0.99	1.09	1.22	1.24
Dietary fiber	—	—	—	—	17.94	17.52	17.54	13.37
Total polyphenol	—	—	—	—	0.04	0.02	0.14	0.05

1 Values are g/100 g of legume. Dietary fiber and polyphenols were only measured in cooked legumes. CP, chickpea; DHMB, dehulled mung bean; IAA, indispensable amino acid; MB, mung bean; YP, yellow pea.

The true mean ileal IAA digestibility for chickpea, yellow pea, whole mung bean, and dehulled mung bean are provided in **Tables 4** and **5**. It was highest for chickpea at 75% and lowest for whole mung bean at 63%. The true mean ileal IAA digestibility of each legume was significantly different from each other (chickpea versus yellow pea, chickpea versus whole mung bean, and yellow pea versus whole mung bean, all *P* < 0.005). Dehulling improved the mean ileal IAA digestibility of mung bean by 8% (*P* < 0.001), and this increase ranged from 0.4% for lysine to 12.1% for valine. In the experiment that tested the validity of using a standard of intact protein ([U-^13^C] spirulina) of known digestibility, the true mean ileal IAA digestibility of whole mung bean, remeasured against a standard of a ^13^C-algal IAA mixture, was very similar to whole mung bean digestibility measured against the [U-^13^C] spirulina standard (64% versus 63%; *P* > 0.05).

**TABLE 4:** True ileal digestibility values of IAA from chickpea, yellow pea, and mung bean in apparently healthy Indian adults^1^

	CP^2^	YP^2^	MB^2^
Amino acids	(*n* = 6)	(*n* = 6)	(*n* = 6)
Methionine	71.8 ± 3.9^a^	56.1 ± 2.8^b^	52.2 ± 7.2^b^
Phenylalanine	80.9 ± 2.5	80.6 ± 1.6	73.4 ± 6.3
Threonine	72.5 ± 2.7^a^	66.8 ± 5.7^a^	42.5 ± 1.2^b^
Lysine	60.0 ± 3.0	62.1 ± 5.7	63.0 ± 5.4
Leucine	79.5 ± 2.6^a^	79.0 ± 1.3^a^	67.5 ± 3.2^b^
Isoleucine	81.4 ± 2.0	79.5 ± 4.6	75.8 ± 2.6
Valine	75.9 ± 3.0^a^	77.4 ± 1.6^a^	67.8 ± 6.0^b^
Mean IAA	74.6 ± 0.8^a^	71.6 ± 1.3^b^	63.2 ± 1.5^c^

1 Values are means ± SDs. CP, chickpea; IAA, indispensable amino acid; MB, mung bean; YP, yellow pea.

2 Means not sharing a common superscript letter in the same row are significantly different at *P* < 0.05 based on ANOVA and a Bonferroni correction.

**TABLE 5:** True ileal digestibility values of IAA from mung bean and dehulled mung bean in apparently healthy Indian adults^1^

	True ileal digestibility (%) of the test protein group		
	MB^2,3^	MB-^13^CAA^2,3^	DHMB^2^
Amino acids	(*n* = 6)	(*n* = 5)	(*n* = 6)
Methionine	52.2 ± 7.2	48.7 ± 6.3	64.3 ± 4.7
Phenylalanine	73.4 ± 6.3	74.6 ± 1.4	75.1 ± 3.0
Threonine	42.5 ± 1.2	42.7 ± 3.2	54.5 ± 2.4^a^
Lysine	63.0 ± 5.4	69.3 ± 3.4	63.4 ± 3.6
Leucine	67.5 ± 3.2	69.3 ± 5.0	76.3 ± 3.2^a^
Iso-leucine	75.8 ± 2.6	76.6 ± 5.0	82.9 ± 3.0
Valine	67.8 ± 6.0	66.7 ± 5.1	80.0 ± 3.2^a^
Mean IAA	63.2 ± 1.5	64.0 ± 2.4	70.9 ± 2.1^a^

1 Values are means ± SDs. DHMB, dehulled mung bean; MB, mung bean; MB-^13^C AA, mung bean true ileal digestibility referenced to standard ^13^C IAA mixture. Subjects of the MB-^13^CAA study were a subset of the subjects of the MB study.

2 Paired t test between MB versus DHMB (*n* = 6) and MB versus MB-^13^CAA (*n* = 5).

3 No significant difference between MB versus MB-^13^CAA.

a True ileal mean and individual IAA digestibility significantly higher than mung bean IAA digestibility (*P* < 0.05).

Among the legume protein groups, the true ileal IAA digestibility ranged between 42% for threonine and 83% for isoleucine. The lowest and highest digestibility values differed between the test protein groups; for chickpea they ranged from 60% for lysine to 81% for isoleucine, for yellow pea from 56% for methionine to 81% for phenylalanine; for whole mung bean from 42% for threonine to 76% for isoleucine, and for dehulled mung bean from 55% for threonine to 83% for isoleucine. When a ^13^C-algal IAA mixture was used as the standard to measure the digestibility of whole mung bean, this ranged from 43% for threonine to 77% for isoleucine, which was similar to the digestibility measured by the intact [U-^13^C] spirulina standard. The CV for true ileal IAA digestibility among the legumes ranged between 1.7% for leucine and 13.8% for methionine. Specifically, for chickpea the CV ranged between 2.5% (isoleucine) and 5.4% (methionine), for yellow pea it ranged between 1.7% (leucine) and 9.1% (lysine), and for whole mung bean it ranged between 2.7% (threonine) and 13.8% (methionine). For dehulled mung bean, the CV ranged between 3.6% (isoleucine) and 7.3% (methionine).

## Discussion

This study provides the true mean ileal IAA digestibility of commonly consumed legumes in healthy Indian adults. The true mean ileal IAA digestibilities of *kabuli* chickpea, yellow pea, and whole and dehulled mung bean were found to be 75%, 72%, 63%, and 71%, respectively. Overall, the digestibility values of threonine, lysine, and methionine were low in all the legumes studied when compared with the other IAA. This is relevant since the products of the individual IAA digestibilities and their concentrations define protein quality. Methionine with its lower concentration and digestibility was the limiting amino acid in the legumes studied. The true mean ileal IAA digestibility values of the present study were compared with digestibility values of either protein or IAA estimated with different digestibility protocols in rat, pig, and human models where available (**Table 6**). The mean true ileal IAA digestibility of boiled *kabuli* chickpea from this study was about 10% lower than the true ileal IAA digestibility of chickpea curry or the true orofecal nitrogen and IAA digestibility of cooked *kabuli* and canned chickpea determined in rats (6, 7, 17). For yellow pea, the mean true ileal digestibility of this study was lower (by 18% and 19%, respectively) than that of green pea and pea protein isolate in humans (18–20), as well as pea protein concentrate in pigs (21). However, the present study values compared well with the true orofecal IAA digestibility of soaked and autoclaved yellow pea (77.9%) in rats (8). The processing of legume in the latter study was quite similar to that in the present study, and all individual IAA digestibility values were similar except the value for lysine. The mean true ileal IAA digestibility of whole mung bean in the present study was lower by about 20% than the standard ileal IAA digestibility and true nitrogen digestibility determined in pigs and rats for steamed and boiled mung bean, respectively (22, 23). This study also showed an improvement in the mean true ileal IAA digestibility of mung bean by 8% on dehulling. By comparison, dehulled mung bean digestibility was lower by about 20% when compared with the true ileal IAA and orofecal nitrogen digestibility in rats (17, 23).

**TABLE 6:** Comparison of digestibility values from adult humans or animal models using different methods of preparation and digestibility measurement with values from this study for the 4 test proteins

Legume protein	Animal model	Type of legume	Method of preparation	Digestibility measurement method	Mean digestibility value	Reference
Chickpea	Human	*Kabuli*	Soaked for 12 h and pressure cooked	Dual-isotope technique	74.6	Present study
	Rat		Curry	True ileal AA digestibility	86.3^2^	(17)^3^
		*Kabuli*	Soaked for16 h and cooked for 22.7 min	True orofecal N digestibility	85.0	(6)
		Canned	Dried	True orofecal AA digestibility	85.2^2^	(7)^3^
		Canned		True orofecal AA digestibility	86.0^2^	(24)^1,3^
Yellow pea	Human	Whole	Soaked for 12 h and pressure cooked	Dual-isotope technique	71.6	Present study
		Whole (green peas)	Ground and boiled at 100°C for 1 h	True oroileal N digestibility	89.4	(18, 19)
		Protein isolate	Finely ground and protein isolated	True oroileal N digestibility	89.9	(20)
	Pig	Protein concentrate		Standard ileal AA digestibility	90.6	(21)^1^
	Rat	Dehulled	Cooked for 37 min	True orofecal N digestibility	87.9	(6)
		Whole	Soaked for 18 h and autoclaved for 10 min	True orofecal AA digestibility	77.9^2^	(8)
Whole mung bean	Human	Whole	Soaked for 12 h and pressure cooked	Dual-isotope technique	63.2	Present study
	Pig	Whole	Steamed	Standard ileal AA digestibility	83.6^2^	(22)
	Rat	Whole	Boiled for 30 min	True orofecal N digestibility	83.7	(23)
Dehulled mung bean	Human		Soaked for 12 h and pressure cooked	Dual-isotope technique	70.9	Present study
	Rat		Curry	True ileal AA digestibility	90.1^2^	(17)
			Dehulled after toasting and cooked for 20 min	True orofecal N digestibility	81.6	(23)
			Dehulled mechanically and cooked for 10 min		97.1	

1 Cooking method not mentioned.

2 Values are mean of IAAs as reported in the present study.

3 Variety not mentioned.

The lower values in humans may be due to differences in the way the legume was processed, the food matrix, and species-specific differences (discussed below). However, the aim here was to measure digestibility of commonly consumed legumes in meal preparations that were representative of habitual consumption. For instance, the difference in the method of dehulling and cooking time of mung bean showed a difference of 16% in true orofecal nitrogen digestibility in rats (23). Equally important is the food matrix and the particle size of the test protein (25); this provides an additional dimension to the variability in the digestibility measurements. This is evident in rat protein digestibility studies where a ground test protein is administered (6–8) and where the digestibility tends to be higher than in human or pig studies (26). The variety of legume is also important as the true mean ileal digestibility of *desi* chickpea, native to South India (12), was about 15% lower than that of the *kabuli* chickpea variety measured in this study. The type of processing the food has undergone can affect digestibility (27). While there were significant differences in the mean IAA digestibility between legumes, the true ileal digestibility of lysine was low and similar across the legumes at about 60%, including the dehulled mung bean. This may be due to loss of available lysine during cooking by formation of Maillard-like products following reaction with reducing sugars, thus leading to unavailability for metabolic utilization (17, 28), since all the legumes underwent a similar cooking protocol. Further, the true ileal lysine digestibility of mung bean increased in the validity experiment with the ^13^C-IAA mixture as standard, compared with that of [U-^13^C] spirulina; this difference was within the variation of the amounts of available lysine that could result from cooking, even though this process was the same throughout (29).

Matrix effects, including the presence of components in the meal which may interfere with the intestinal breakdown of proteins, can also affect digestibility. In this context, the lower true ileal IAA digestibility values of legumes when compared with those for ASF (9) can be attributed in part to the presence of ANFs such as phytic acids, polyphenols, tannins, hemagglutinins, anthocyanidins, trypsin, and protease inhibitors (13, 27). Another major factor that plays an important role in decreased digestibility of plant proteins is the presence of intact cell walls (30). Protein digestibility can be increased by reducing the amount of anti-nutrients and breaking down the cell wall in the foods. Soaking, dehulling, germination, fermentation, milling, and pressure cooking are the most commonly used processing techniques that improve digestibility by reduction of ANFs and breakdown of cell walls (13, 31). Each processing technique reduces a particular anti-nutrient more effectively than the other. For instance, dehulling of faba bean and broad bean reduced their tannin and polyphenol content by 90%, whereas it had no effect on the phytic acid content. However, germination of the same legumes decreased phytic acid by 60% and 30%, respectively (32). Therefore, a combination of processing techniques can be used to achieve a higher digestibility of legumes. This is relevant, as it has been shown that the digestibility of soy protein can be increased by processing to match that of ASF (33). A final consideration is the structure of the dietary proteins and their effect on protein digestion, where it has been demonstrated that the proteins in the common bean and faba bean on thermal treatment form insoluble aggregates which escape digestion (34).

The dual-isotope tracer method has been described as a potentially useful minimally invasive method that could be used in humans (10) and was used first in humans to determine the digestibility of phenylalanine (35) or different IAAs in spirulina and legume protein (12). The measurement of digestibility by this method is based on the plasma appearance, or isotopic enrichment, of the 2 isotopically (^2^H and ^13^C) labeled IAAs from the test and standard protein, respectively, which are normalized to their isotopic enrichment in the meal (12). The method is independent of splanchnic handling of IAA, since differently labeled IAA released from the test and standard protein will have similar metabolic and recycling fates (assuming minimal isotopic effects) and therefore the digestibility measurement is independent of first-pass metabolism (10). The method does not require the additional measurement of endogenous protein losses (10). This study also validated the use of ^13^C-labeled, commercially available, intact protein (spirulina) as a standard protein, by comparing the digestibility of whole mung bean when measured against the spirulina standard or a free IAA mixture; there were no significant differences in ileal IAA digestibility by either method.

The true ileal IAA digestibility of each legume will inform the DIAAS. These DIAAS values can be used in turn to optimize lysine-limited cereal-based diets that are usually eaten by poor populations with high-quality protein sources, especially in growing children, with similar (65%) ileal lysine digestibility of legumes (36), which means that the addition of legumes to such diets should not be calculated based on their protein content alone. In cereal-based diets, proportionately more legumes would be needed to meet the quality protein requirement in comparison to other high-quality protein sources such as ASF (36). The EAT–Lancet Commission food plate, recommended for environmental sustainability while meeting macro- and micronutrient requirements for population health, defined a combination of reduced proportions of ASF and increased PSF (5). However, the definition of sustainability can vary regionally (37). Furthermore, environmental costs of ASF should be considered individually because milk, eggs, and poultry have lower environmental impact than beef (4). Additionally, though such a diet is optimal for the daily quality protein requirement, for a significant proportion of vegetarian populations (38), replacing ASF would require a considerably higher quantity of legumes, owing to their low digestibility. Then, shifting to more sustainable cereal and legume-based agricultural practices needs to be linked to the appropriate complementarity of mixed foods, as well as improved postharvest technologies or home-based food processing, which could reduce ANF and improve protein digestibility.

The strength of this study is the use of intrinsically labeled legumes to determine their true ileal IAA digestibility in healthy human adults. A possible limitation is the use of previously determined digestibility values of the standard (spirulina) protein in different individuals; however, this was validated for mung bean digestibility, which remained similar when remeasured against a standard of free IAA in the same subjects. Possible limitations of the method are the assumption that the free amino acids are completely absorbed and the absorbed amino acids from the test and standard undergo similar first-pass metabolism and intestinal recycling. Additionally, since the dual-isotope technique is relatively new, there is now a need to validate it against methods which measure digestibility at the end of the ileum. In conclusion, this study provides the true ileal IAA human digestibility of 3 commonly consumed legumes in typical meals and demonstrates the improvement in mung bean digestibility by dehulling.

## Supplementary Material

nqz159_Supplemental_File

## Notes

SK and AV contributed equally to this work.

This research work was supported by The Wellcome Trust/DBT India Alliance through the Margdarshi fellowship to AVK, and partly by IAEA CRP Code: E4.30.31, contract no: 19301.

Supplemental Figures 1 and 2 and Supplemental Tables 1–3 are available from the “Supplementary data” link in the online posting of the article and from the same link in the online table of contents at https://academic.oup.com/ajcn/.

Abbreviations used: AA, amino acid; ANF, antinutritional factor; ASF, animal source food; DIAAS, digestible indispensable amino acid score; ECEE, ethoxycarbonyl ethyl ester; GC-P-IRMS, GC-pyrolysis–isotope ratio mass spectrometry; IAA, indispensable amino acid; LMIC, low- and middle-income countries; ppme, parts per million excess; PSF, plant source food; TCF, transamination correction factor.
