# Supplementary material for: True ileal digestibility of legumes determined by dual-isotope tracer method in Indian adults
Source: Am J Clin Nutr. 2019 Aug 2;110(4):873–82. doi: 10.1093/ajcn/nqz159 (PMC6766447; doi:10.1093/ajcn/nqz159)
Supplement: nqz159_Supplemental_File [file nqz159_supplemental_file.pdf]

Supplemental Figure 1. Subject screening and enrolment details

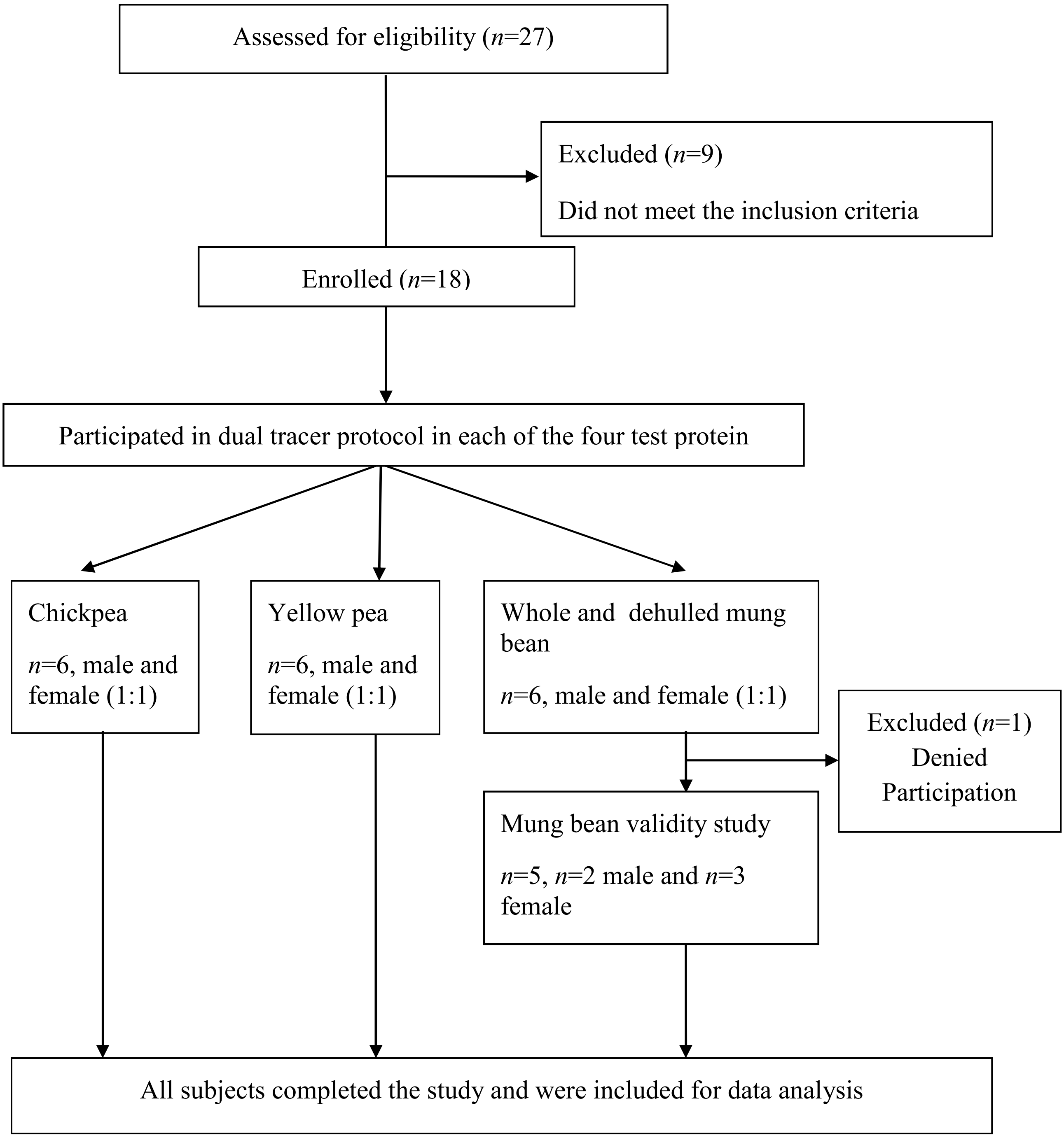

Supplemental Figure 2. Breath $^{13}CO_2$ enrichment after consumption of chickpea, yellow pea, whole and dehulled mung bean test meals by healthy Indian adults. Expressed in atom percent (At%); represented as mean ± SD

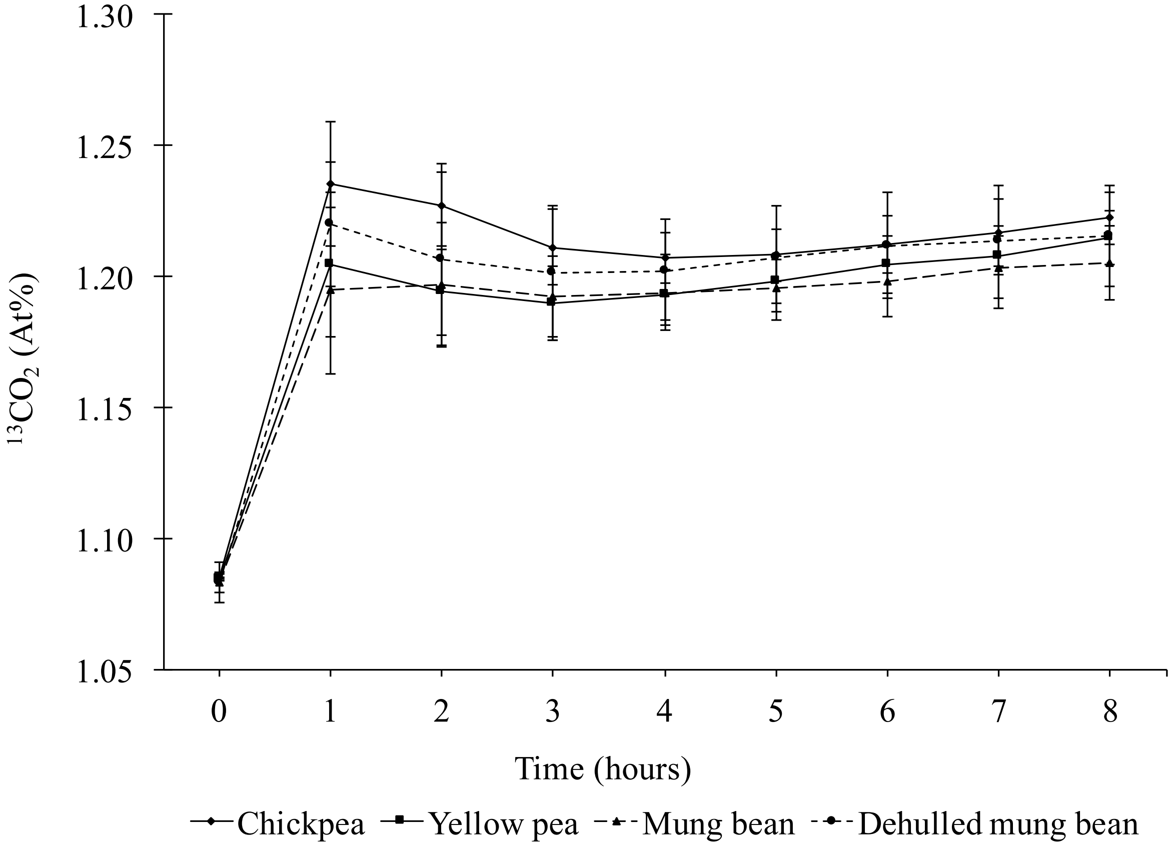

Supplemental Table 1. Composition of ^{13}C algal amino acid mixture[1]

Amino Acids	Concentration (%)
Aspartate	8.8
Threonine	3.2
Serine	4.6
Glutamate	8.2
Proline	4.0
Glycine	8.3
Alanine	11.9
Valine	7.0
Methionine	2.0
Isoleucine	6.0
Leucine	12.2
Tyrosine	3.9
Phenylalanine	5.4
Histidine	1.2
Lysine	5.8
Arginine	5.9

[1]Reproduced from certificate of analysis of Sigma Aldrich, MO, USA

Product name: Algal amino acid mixture-^{13}C-98 atom% ^{13}C

Supplemental Table 2. ^{2}H enrichments in pooled samples of intrinsically labelled chickpea, yellow pea and mung bean seeds expressed as parts per million excess (ppme)[1]

Amino Acids	Pooled CP	Pooled YP	Pooled MB
Methionine	236	312	565
Phenylalanine	710	911	1611
Threonine	998	862	2426
Lysine	578	589	1169
Leucine	816	866	1843
Isoleucine	750	873	1604
Valine	768	803	1519
Mean	694	745	1534

[1]Values are parts per million excess (ppme); CP, Chickpea; YP, yellow pea; MB, mung bean; DHMB, de-hulled mung bean.

Supplemental Table 3. Meal enrichments (ppm excess*10^3) of ^2H and ^{13}C test meal of chickpea, yellow pea, whole and dehulled mung bean.[1,2]

Amino Acids	CP		YP		MB		MB-^{13}CAA		DHMB	
	^2H	^{13}C	^2H	^{13}C	^2H	^{13}C	^2H	^{13}C	^2H	^{13}C
Methionine	0.16 ± 0.02	0.49 ± 0.06	0.17 ± 0.02	0.45 ± 0.07	0.15 ± 0.05	0.48 ± 0.22	0.34 ± 0.01	0.19 ± 0.04	0.14 ± 0.06	0.27 ± 0.06
Phenylalanine	0.57 ± 0.07	9.72 ±0.45	0.54 ± 0.03	11.1 ± 0.82	0.46 ± 0.02	12.2 ± 3.01	1.09 ± 0.02	2.81 ± 0.17	0.51 ± 0.25	10.1 ± 0.27
Threonine	0.29 ±0.04	0.47 ± 0.02	0.23 ± 0.03	0.63 ± 0.08	0.53 ± 0.08	0.61 ± 0.09	0.50 ± 0.09	0.15 ± 0.02	0.44 ± 0.15	0.69 ± 0.06
Lysine	0.47 ± 0.04	20.3 ± 0.15	0.42 ± 0.03	19.9 ± 1.75	0.44 ± 0.04	30.1 ± 10.0	0.89 ± 0.02	7.83 ± 0.86	0.44 ± 0.15	15.3 ± 3.04
Leucine	0.61 ± 0.06	0.80 ± 0.02	0.54 ± 0.02	0.95 ± 0.06	0.58 ± 0.02	1.03 ± 0.18	1.21 ± 0.04	0.09 ± 0.01	0.64 ± 0.23	0.65 ± 0.16
Iso-leucine	0.54 ± 0.02	0.83 ± 0.09	0.53 ± 0.01	0.92 ± 0.08	0.51 ± 0.01	1.05 ± 0.33	1.03 ± 0.03	0.07 ± 0.003	0.52 ± 0.20	0.57 ± 0.13
Valine	0.52 ± 0.05	0.57 ± 0.05	0.47 ± 0.02	0.73 ± 0.06	0.49 ± 0.02	0.66 ± 0.15	0.93 ± 0.05	0.06 ± 0.008	0.52 ± 0.17	0.48 ± 0.14

[1]Values are mean ± SD (ppme*10^3); CP, chickpea; YP, yellow pea; MB, mung bean; MB-^{13}CAA, mung bean true ileal digestibility referenced to standard ^{13}C indispensible amino acid mix; DHMB, dehulled mung bean.

[2]Subjects were different in CP, YP, MB digestibility studies (*n*=3 each male and female). Paired studies for MB vs. DHMB (*n*=6) and MB vs. MB-^{13}CAA (*n*=5, *n*=3 female and *n*=2 male). Subjects of MB-^{13}CAA study were sub-set of MB study.
